# Mitigation of efflorescence of wallboard by means of bio-mineralization

**DOI:** 10.3389/fmicb.2015.01155

**Published:** 2015-10-20

**Authors:** Bin Xue, Chunxiang Qian

**Affiliations:** ^1^School of Materials Science and Engineering, Southeast UniversityNanjing, China; ^2^Research Institute of Green Construction Materials, Southeast UniversityNanjing, China

**Keywords:** wallboard, bio-mineralization, *Bacillus mucilaginosus*, carbon dioxide, efflorescence

## Abstract

Cement-based material is one of the most versatile and largest amounts of building materials which can not only be used in load-bearing structure but also be used as decoration materials, like brick, wallboard, and tile. However, white calcium carbonate always be found on the surface of wallboard. This phenomenon is generally called efflorescence, which has no damage to wallboard, but has aesthetic impact. In this research, *Bacillus mucilaginosus* was pre-added to the cement matrix to reduce the efflorescence of wallboard. Image processing, thermogravimetric analysis and permeability test were used to characterize the efflorescence degree of wallboard. The results showed that the bacterium captured atmospheric CO_2_ by carbonic anhydrase and promoted the CO_2_to react with Ca(OH)_2_. This process not only reduced the content of Ca(OH)_2_ but also improved the compactness of wallboard. In addition, the maximal decrease of efflorescence area of wallboard was gotten when the content of microbial was up to 4% of the mass of cementitious material and the proportion of efflorescence area reduced from 32 ± 3 to 5 ± 1% of the whole area of surface layer. At the same time, the values of compressive and flexural strength were the highest and the surface layer of wallboard was the most compact. The observed reduction of efflorescence was indeed due to the effect of bio-mineralization. This promising method was noted to be cheap, convenient, environment friendly, and which has the potential in various practical applications.

## Introduction

With the increasingly high demand for housing decoration, the diversity of the shape of wallboard was pursued, and the aesthetics of wallboard was also noticed. However, most wallboard is made of cement based materials which can cause efflorescence. The materials of efflorescence are usually white and water-soluble compounds which deposit on the surface of masonry (ASTM C1400-11, 2011). Mostly, whitish deposits of water soluble salts are alkali sulfate or sodium chloride, which generally appear soon after erection of the facade. In addition, the appearance of white efflorescence depends on environmental circumstances, material parameters of bricks, mortar and concrete blocks, and may additionally be influenced by treatments of the façade (Brocken and Nijland, 2004; Chwast et al., 2015). According to the existing researches, a physicochemical model was developed to explain the key features and the formation of efflorescence (Kresse, 1982, 1987; Vickers and Moukwa, 1996; Dow and Glasser, 2003). It was suggested that the appearance of efflorescence is due to the low compactibility of cement based materials.

Efflorescence is widely known as an aesthetic problem of brick and block masonry facades. However, up to now little pragmatic remedy has been found for this phenomenon. Nowadays, the common approach to reduce the efflorescence of wallboard is adding active mineral admixture into cement matrix. This way has the characteristic of high cost. The main objective of this study was to investigate the effects of bio-mineralization on efflorescence of wallboards. Bio-mineralization is a new technology which has been used in many fields like heavy metal treatment, concrete crack repairment and so on. Van Tittelboom (Van Tittelboom et al., 2011) used urease bacteria which can induce CaCO_3_ deposition to repair cracks on the surface of concrete. Wang (Wang et al., 2013) also utilized bacteria to repair cracks of concrete and the maximal width reached 970 μm. Rodriguez-Navarro (Rodriguez-Navarro et al., 2003) induced calcium carbonate precipitation on the surface of porous limestone by myxococcus xanthus and found that the thickness of mineralized layer was up to 10–50 μm. Bundur (Bundur et al., 2015) investigated the effect of *Sporosarcina pasteurii* on the mortar and she found that the content of calcium carbonate increased and the content of calcium hydroxide decreased after adding *S. pasteurii* into mortar. She also stated that the microorganism has impact on the compressive and flexural strength of specimens. Some special bacteria can induce carbonate deposition and this process is generally called biomineralization (Mann, 2001). Biomineralization is considered to be an effective microorganism repair strategy and has been extensively researched. This method has been used in many other fields like dust-fixing, foundation reinforcement and so on (Mitchell and Ferris, 2005; Dejong et al., 2006).

*Bacillus mucilaginosus* is a special bacillus species and it can activate some insoluble mineral elements from soil, so this bacterium has been mostly used as biological fertilizer and as a model bacterium in studying microbe-mineral interactions (Lian et al., 2000). Apart from this, this Bacillus can capture atmospheric CO_2_ by carbonic anhydrase (Zhang et al., 2011). The flat colony counting method was adopted to calculate the number of bacterial strain in different pH. The results indicated *B. mucilaginosus* could get maximal activity when the pH of fermentation medium was 8.0 and *B. mucilaginosus* also had good activity in high alkali environment. In this research, a promising way was pre-adding *B. mucilaginosus* to cement matrix to reduce the efflorescence on the surface of cement-based materials. *B. mucilaginosus* contains metalloenzymes, the active center of zinc, so that the bacteria can catalyze interconversion reactions between CO_2_ and HCO${}_{3}^{-}$. The transformation equation is CO_2_ + H_2_O ↔ HCO${}_{3}^{-}$ + H^+^. Image processing technology (Zehnder and Schoch, 2009; Vázquez et al., 2011) was used to intuitively show the efflorescence degree. The optimal amount of *B. mucilaginosus* added into matrix and the effect of the microbe on some mechanical properties of wallboard were studied.

## Materials and methods

### Materials

*B. mucilaginosus* was purchased from China Center of Industrial Culture Collection. Portland pozzolana cement was chosen, and the quartz sand was used as the aggregate. The fineness modulus and average particle size of sand were 2.1–2.6 mm and 0.25–0.35 mm, respectively. The polycarboxylate superplasticizer was purchased from Sika China Company. Steel mold was used to form wallboard and the size of steel mold is 300 × 300 × 30 mm.

Fermentation medium of *B. mucilaginosus* cultivation was shown in Table 1.

**Table 1:** **Purification culture medium c/(g· L^−1^)**.

**Sucrose**	**Na_2_HPO_4_∙12H_2_O**	**MgSO_4_**	**CaCO_3_**	**KCl**	**(NH_4_)_2_SO_4_**	**Yeast Extract**
10.000	2.507	0.2561	1.000	0.100	0.500	0.200

The pH of fermentation medium was adjust to 7 by adding calcium hydroxide solution, and the medium was autoclaved at 121°C for 25 min, then purified strains of *B. mucilaginosus* were inoculated in sterile medium. Then the bacterium was cultured in the shaking incubator in the condition of 30°C and oscillation frequency of 170 r/min for 24 h. In order to grasp the growth law of *B. mucilaginosus*, the complete growth curve of *B. mucilaginosus* should be drawn. The initial pH of culture medium was adjust to seven and the optical density of bacteria liquid was measured every 2 h by multifunctional microplate reader (OD) with wavelength of 400 nm.

**Figure 1:**
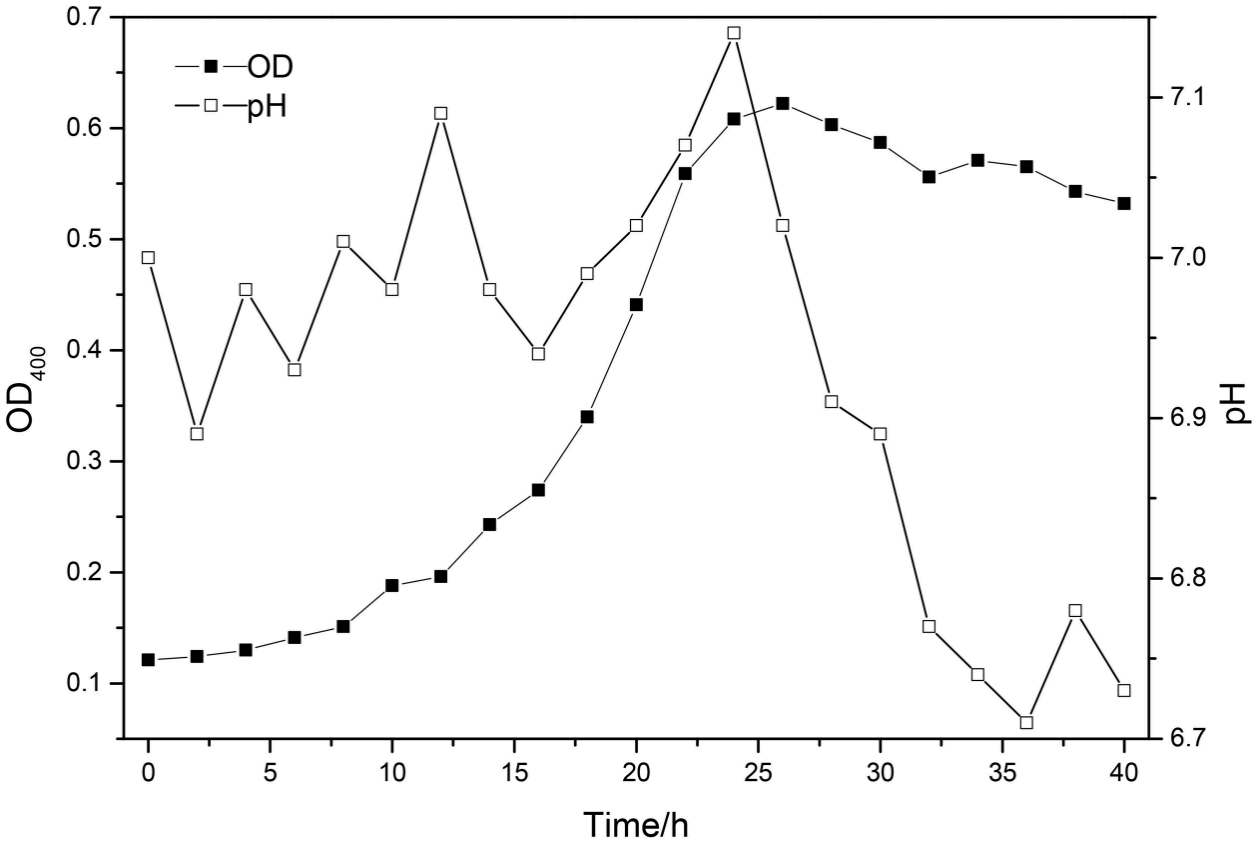
**The growth curve of *Bacillus mucilaginosus* at 25°C**.

The growth curve of *B. mucilaginosus* was shown in Figure 1.

As indicated by the above test: the best time of culturing *B. mucilaginosus* should be 24–28 h. The growth of *B. mucilaginosus* was stable in this period and the total number of bacteria and metabolites reached maximum when the time of culturing *B. mucilaginosus* was 24–28 h.

### Image processing by means of ImageJ

#### Preparation of samples

All samples were prepared by the standard steel model with 300 × 300 × 30 mm. The sand to cement ratio (s/c) for all mortars were 1.0 (w/w). 1200 g Portland pozzolana cement (20% natural pozzolans), 1200 g sand, 369.12 g water, and 4 g water reducing agent were mixed and stirred by mortar mixer. Different content of *B. mucilaginosus* was added in the process of stirring. Then the mixture was poured into the steel model. In this research, seven samples were prepared with different content (0%, 1%, 2%, 3%, 4%, 5%, 6%) of *B. mucilaginosus* respectively. Duplicate samples were prepared for analyzing.

#### Experimental procedure

**Figure 2:**
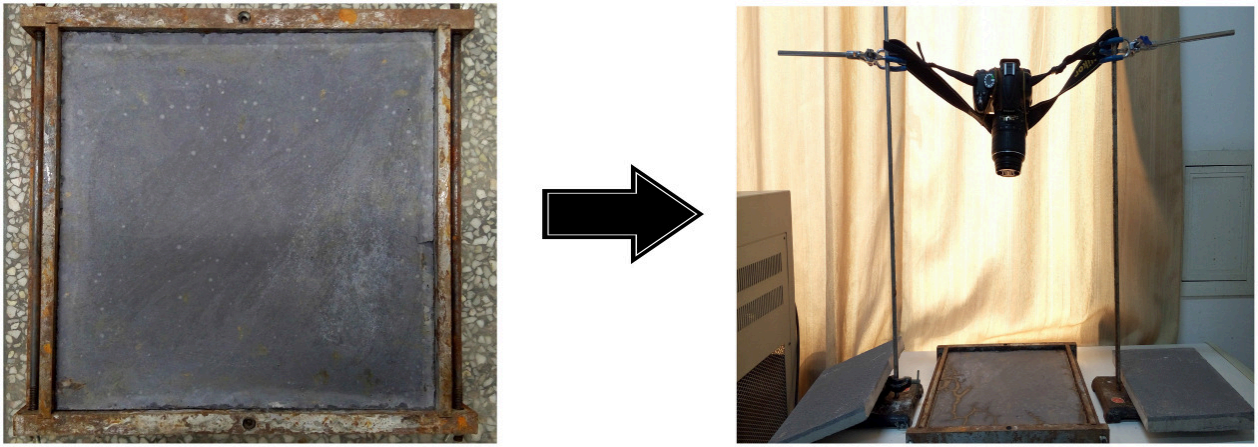
**The digital image processing**.

Prepared samples were placed in the laboratory at 20 ± 3°C for 1 day and then were covered with a layer of water. The thickness of water layer above the surface of wallboard was about 2–3 mm. The water layer disappeared about 2 days later at 20 ± 3°C. Repeat the above process for three times. This approach brought the internal soluble components into the surface of wallboard. So this process was to simulate rain erosion phenomenon and provoke efflorescence. Seven days later, all samples were taken photographs in the condition of same height and same light source by digital camera (Canon 5D Mark III) and the Imagej was used to analysis the surface efflorescence of wallboard. The digital image processing could be revealed by Figure 2.

### Thermogravimetric analysis

#### Preparation of samples and experimental procedure

The production process of wallboard was same like 2.2.1. Seven wallboard samples with different content of *B. mucilaginosus* were placed in laboratory at 20 ± 3°C for 1 day and then the molds were removed. Then surface layer (about 1–2 mm) of wallboard was ground into powder form which was placed in oven for drying away water. The samples were kept in a vacuum desiccator for 24 h and then placed in the TGA analyzer. This testing was conducted by increasing the temperature from 50 to 1200°C. The heating rate was set as 20°C/min and N_2_ was used as shielding gas. Decomposition of calcium hydroxide was determined between 450 and 550°C, and calcium carbonate decarbonation was measured between 700 and 900°C (Alarcon-ruiz et al., 2005). TGA analyzer (FEI Company, Netherlands) was used for the analysis and each sample was tested in duplicate.

### Permeability, compressive, and flexural strength

#### Preparation of samples and experimental procedure

Mortar samples were prepared with the standard of JGJ/T70-2009. It is the test method of basic properties of construction mortar^1^. Seven types of mortars were prepared with different content of *B. mucilaginosus* (0, 1, 2, 3, 4, 5, and 6%) respectively. All the proportion of raw materials for molding wallboard was same like 2.2.1. In the permeability experiment, the mortars were cast into conical bottomed metal mold (the diameter of the upper opening was 70 mm, outlet diameter was 80 mm, and the height was 30 mm). These specimens were cured at room temperature (25 ± 3°C) for 24 ± 2 h. Then the molds were removed, and specimens were placed in curing room at 20 ± 2°C and relative humidity of 50% for 1, 7, and 28 days. The permeability tests of all specimens were conducted after being placed into mortar permeameter with sealing material. Water permeability was conducted in this experiment and the starting water pressure was 0.2 MPa. If there was no seepage phenomenon on the specimens, then continue increasing water pressure 0.2 MPa until seepage phenomenon happened. The water pressure of starting seepage was recorded. In the compressive and flexural strength test, the mortars were cast into cube molds with 70.7 × 70.7 × 70.7 mm. Then the specimens were removed from the molds after the specimens were placed in curing room at 20 ± 2°C and relative humidity of 50% for 1, 7, and 28 days. Compressive and flexural strength tests were conducted on triplicate samples. The type of flexural strength test was three-point bending experiment.

### SEM images of surface layer of the wallboard

#### Preparation of samples and experimental procedure

The production process of wallboard was same like 2.2.1. The specimens were removed from the molds after all specimens were cured at 25°C and relative humidity of 100% for 7 days. Surface portion was picked out after wallboard was broke into pieces. Then the block was put into the oven to dry away the water before gold sputtering. In order to show the micro structure of the surface layer of wallboard clearly, the SEM images of the hydration products morphology and the interface transition zone of paste-aggregate were displayed. Scanning electron microscope (SEM, FEI Company, Netherlands, operating voltage 20 kV) was used to conduct micro structure studies.

## Results and discussion

### Effects of different content of *B. mucilaginosus* on the efflorescence degree

**Figure 3:**
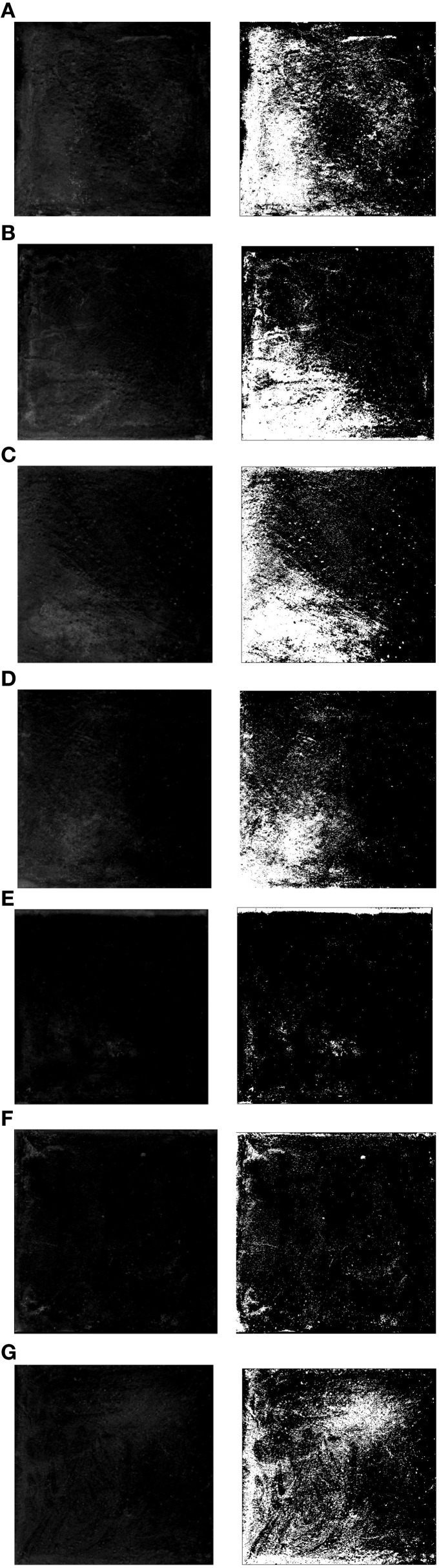
**Efflorescence images of the surface layer with different content of *Bacillus mucilaginosus*. (A)** Bacteria content was 0%, and efflorescence area was 32 ± 3% (control experiment). **(B)** Bacteria content was 1%, and efflorescence area was 28 ± 2%. **(C)** Bacteria content was 2%, and efflorescence area was 26 ± 3%. **(D)** Bacteria content was 3%, and efflorescence area was 17 ± 2%. **(E)** Bacteria content was 4%, and efflorescence area was 5 ± 1%. **(F)** Bacteria content was 5%, and efflorescence area was 10 ± 2%. **(G)** Bacteria content was 5%, and efflorescence area was 18 ± 3%.

Figure 3 showed the efflorescence images processed by means of ImageJ. The experimental results proofed the feasibility of reducing surface efflorescence by *B. mucilaginosus*. However, in the other side, the effect was not always better as the content of *B. mucilaginosus* increased. By calculating with the ImageJ, the efflorescence area was 32 ± 3, 28 ± 2, 26 ± 3, 17 ± 2, 5 ± 1, 10 ± 2, and 18 ± 3% of the whole area of surface layer when the microbe content was 0, 1, 2, 3, 4, 5, and 6% of the mass of cementitious material respectively. In contrast to the efflorescence in the form of lump in Figures 3A–G, the Figures 3E,F both showed the mottled efflorescence on the surface of wallboard. The experimental results could be explained by the mechanism that *B. mucilaginosus* could capture atmospheric CO_2_ by carbonic anhydrase. Then calcium carbonate was produced when CO_2_ reacted with Ca(OH)_2_ (engendered by cement hydration). The generation of calcium carbonate could improve the density of surface layer and reduce the porosity of wallboard. This process could not only reduce the content of Ca(OH)_2_ but also prevented Ca(OH)_2_ from effusing to the surface of wallboard. Efflorescence degree would be increased when microbe content was more than 4% of the mass of cementitious material.

### Effects of different content of *B. mucilaginosus* on calcium hydroxide and calcium carbonate content

**Figure 4:**
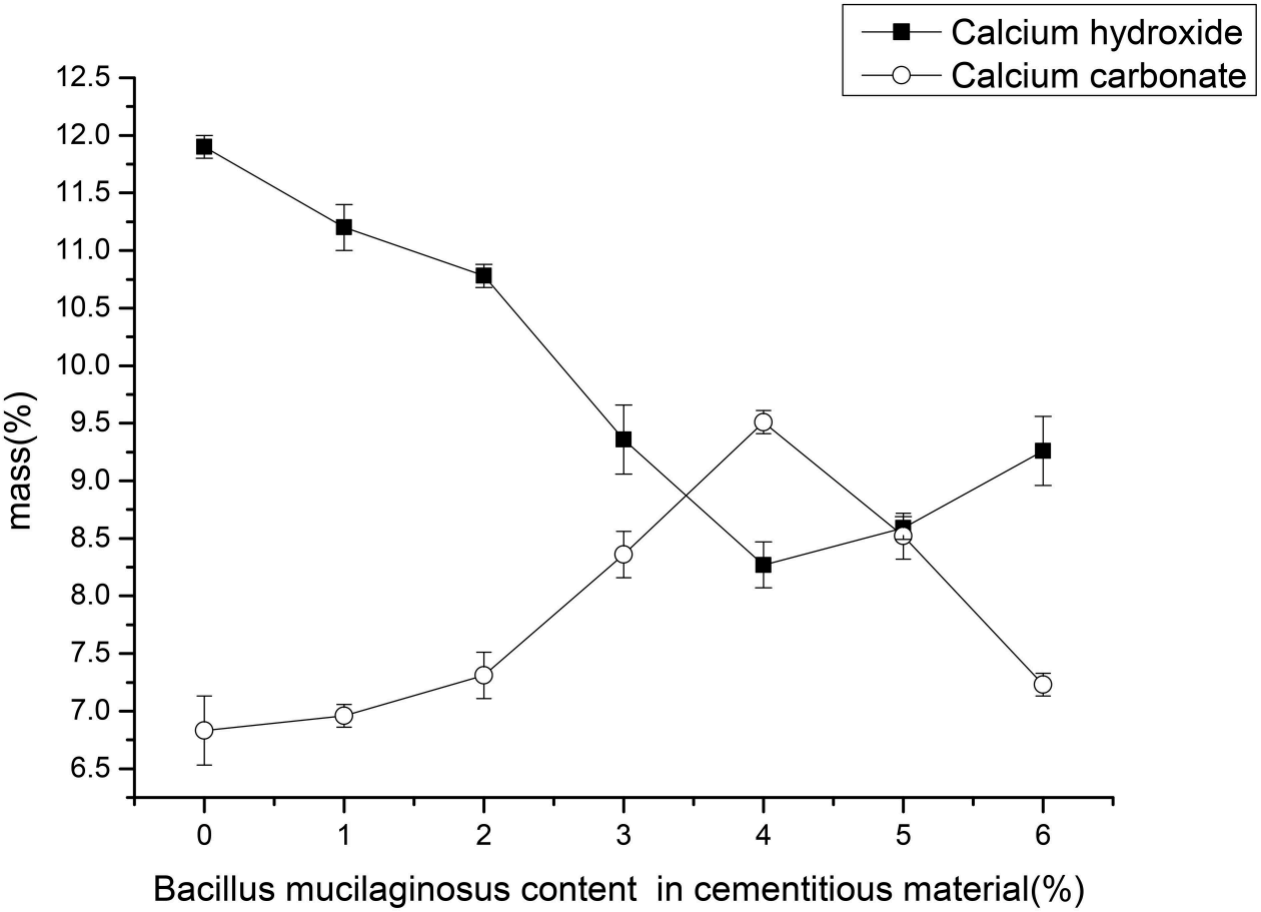
**The content changes of calcium carbonate and calcium hydroxide with the increase of *Bacillus mucilaginosus* content in cementitious material, and error bars represent one standard deviation**.

Figure 4 showed the mass percentages of calcium hydroxide (portlandite) and calcium carbonate of the surface layer of wallboard. The mass percentage of calcium hydroxide (portlandite) was determined by the mass loss between 450 and 550°C, and, similarly, the mass percentage of calcium carbonate was determined by mass loss due to decarbonation between 700 and 900°C. The results showed that mass percentage of calcium hydroxide gradually reduced from 11.90 to 8.27% and calcium hydroxide increased from 6.83 to 9.51% with the increase of *B. mucilaginosus* content from 0 to 4%. However, the mass percentage of calcium hydroxide increased and the mass percentage of calcium carbonate decreased when the content of *B. mucilaginosus* exceed 4%. The experimental results were corresponding with the efflorescence images taken by camera and the mechanism could be explained same like 3.1.

### Effects of different content of *B. mucilaginosus* on permeability, compressive, and flexural strength of wallboard

**Figure 5:**
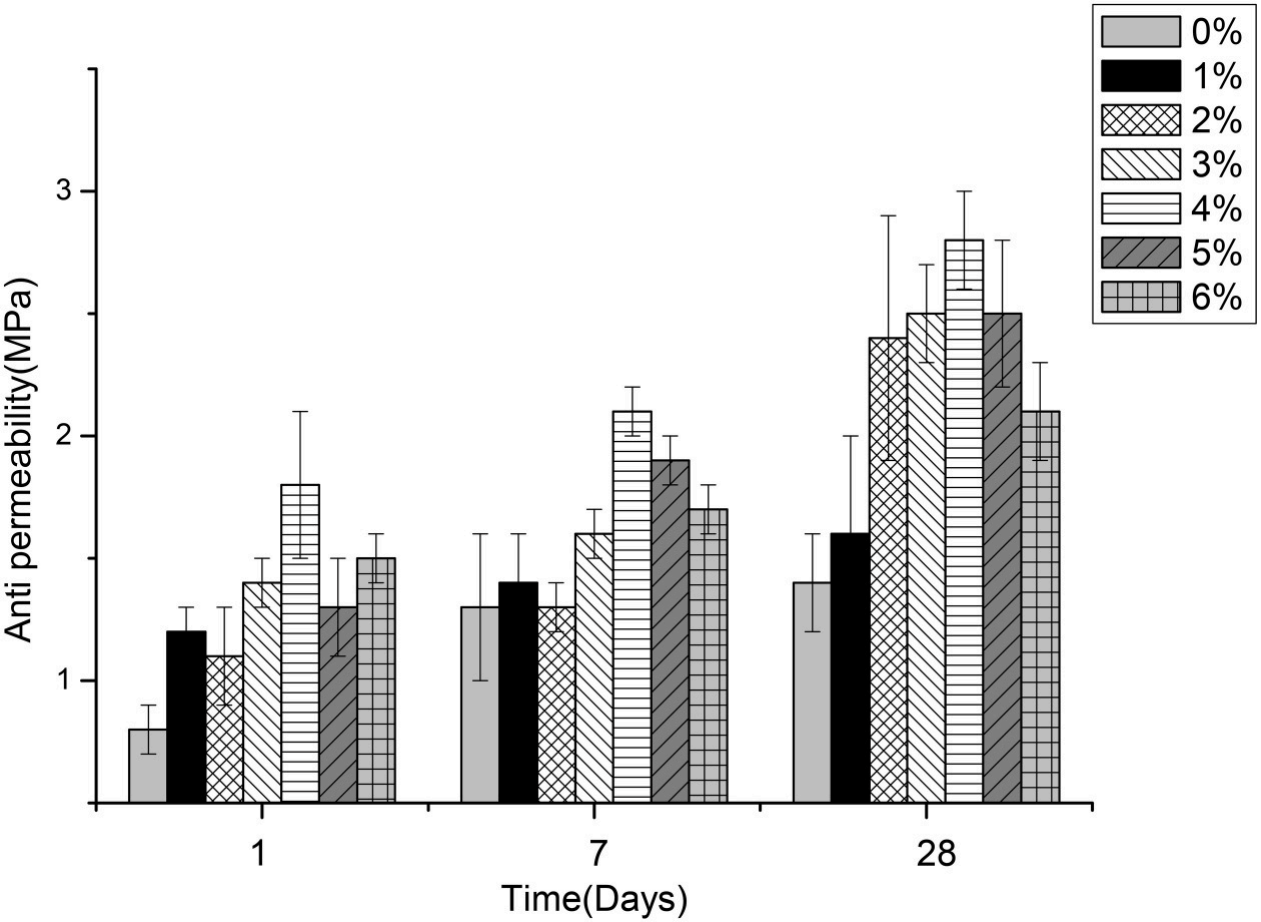
**The changes of permeability with the increase of *Bacillus mucilaginosus* content in cementitious material at 1, 7, and 28 days**. Bars show the average permeability, and error bars represent one standard deviation.

**Figure 6:**
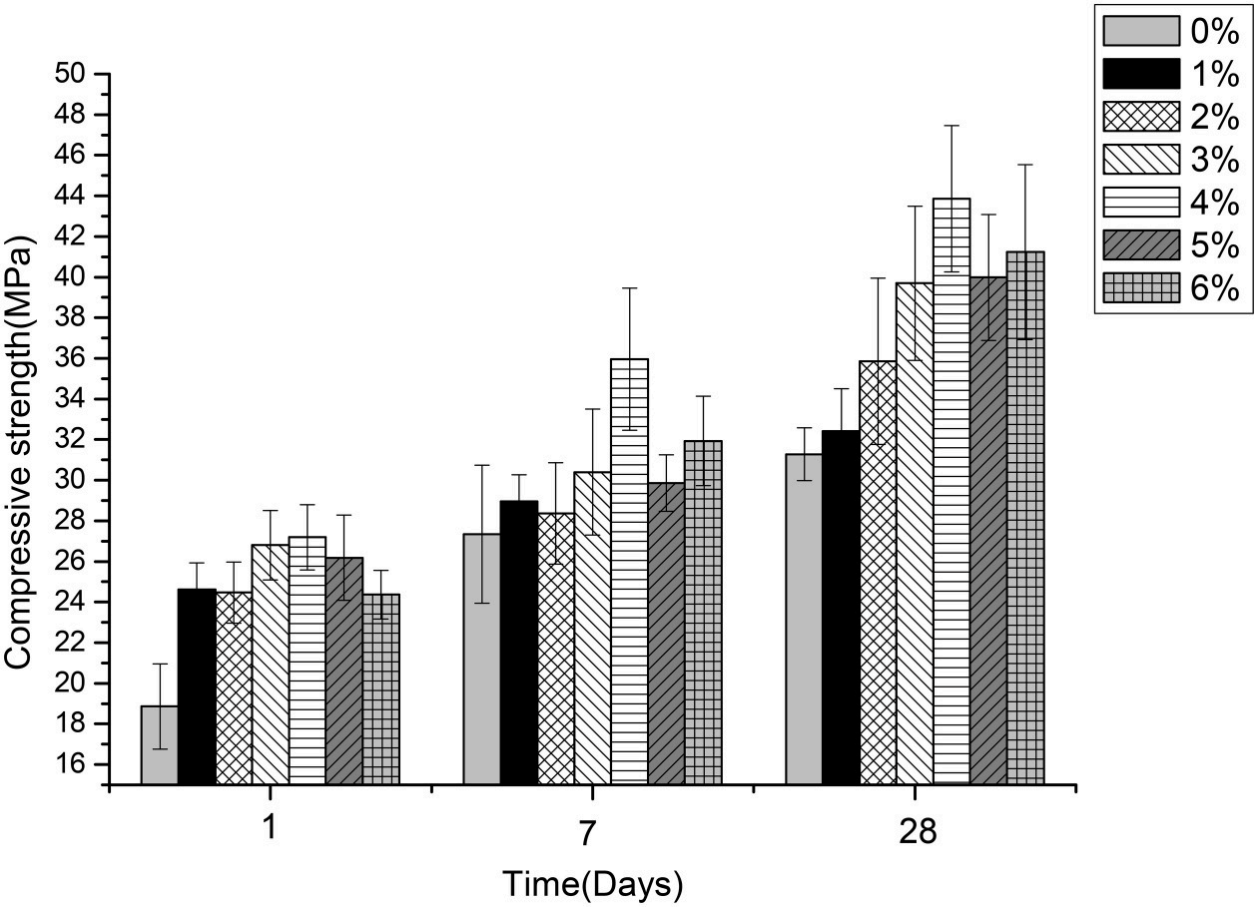
**The changes of compressive strength with the increase of *Bacillus mucilaginosus* content in cementitious material at 1, 7, and 28 days**. Bars show the average compressive strength (based on triplicate mortar samples), and error bars represent one standard deviation.

**Figure 7:**
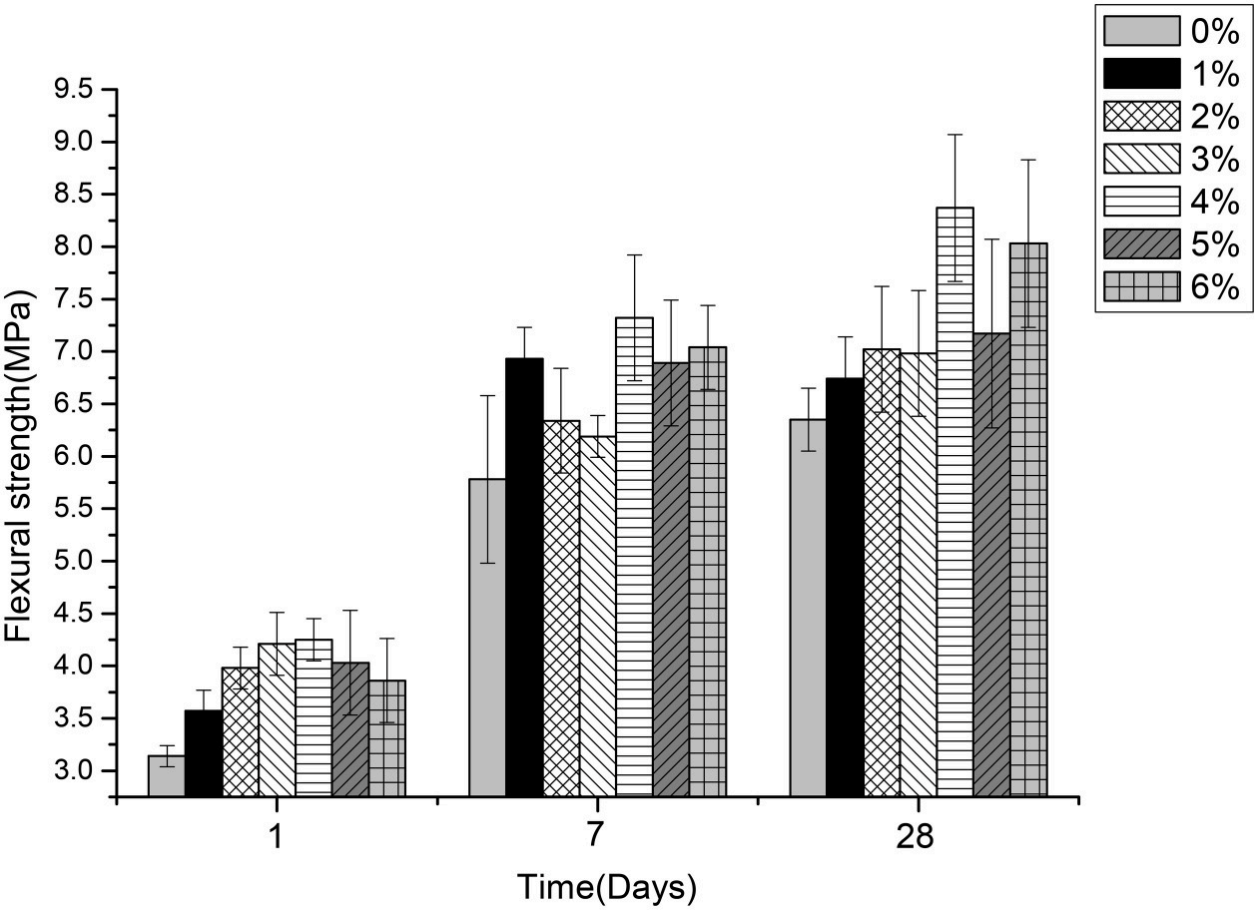
**The changes of flexural strength with the increase of *Bacillus mucilaginosus* content in cementitious material at 1, 7, and 28 days**. Bars show the average flexural strength (based on triplicate mortar samples), and error bars represent one standard deviation.

Figures 5–7 displayed the results of the permeability, compressive, and flexural strength of wallboard, respectively. The permeability, compressive and flexural strength of all the specimens increased with time. At day 1, day 7, and day 28, the specimen always had the best permeability, compressive and flexural properties when the content of *B. mucilaginosus* was 4%. As the content of bacteria was from 0 to 4%, the increase of these properties could be seen. However, these properties decreased when the content exceeded 4%. These phenomena could be contributed to the reduction of porosity owing to microbial induced calcium carbonate formation on the surface layer. The mechanism could also be explained similarly to 3.1. Except that, it also illustrated that the *B. mucilaginosus* could live for a long time in the internal of mortar and the bacteria could absorb CO_2_ from air constantly, which improved permeability, compressive and flexural strength of wallboard greatly at longer ages.

### Effects of *B. mucilaginosus* on micro structure of the surface layer of wallboard

**Figure 8:**
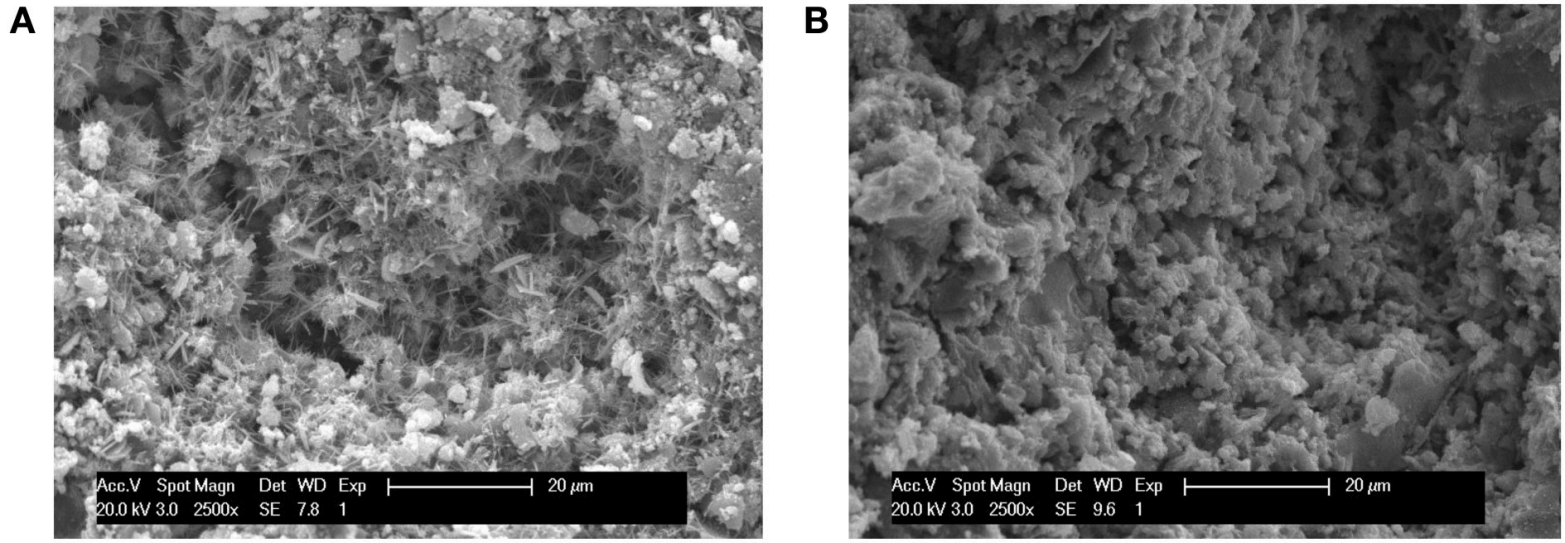
**(A,B)** SEM images of the surface layer of wallboard when the content of *Bacillus mucilaginosus* was 0 and 4%, respectively (The specimens were cured at 25°C for 7 days, and an s/c of 1 was used).

**Figure 9:**
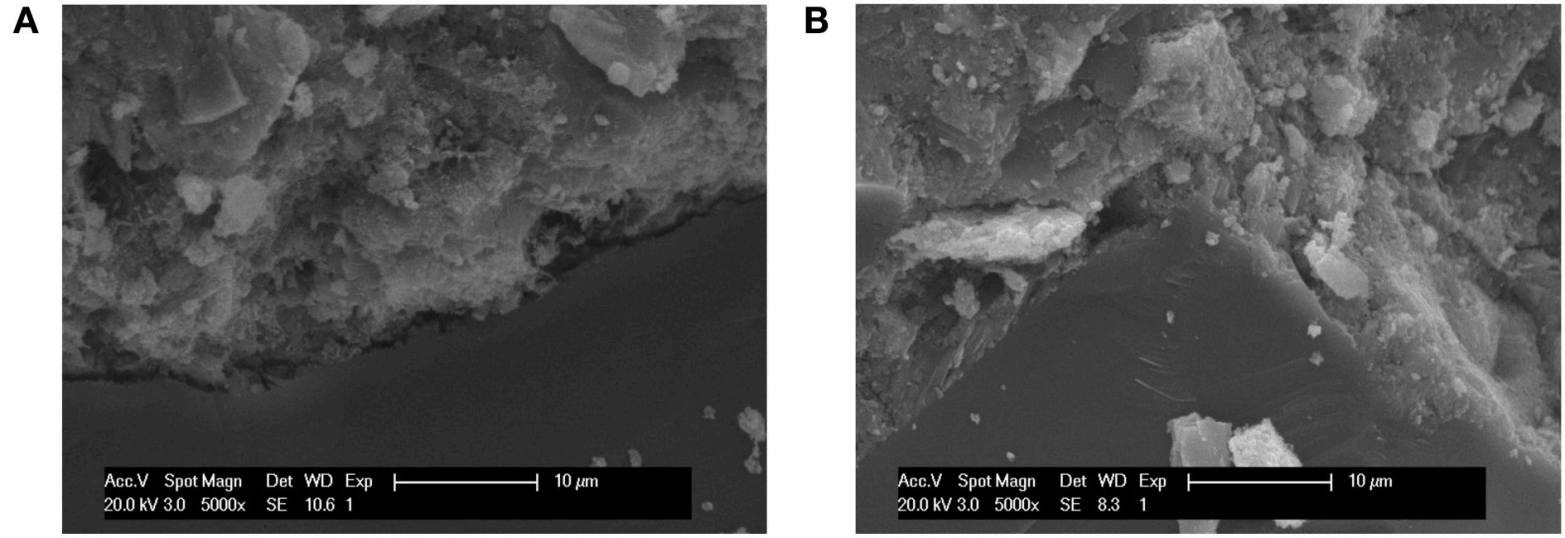
**(A,B)** SEM images of the interface transition zone of paste-aggregate of the wallboard when the content of *Bacillus mucilaginosus* was 0 and 4%, respectively (The specimens were cured at 25°C for 7 days, and an s/c of 1 was used).

Figure 8 showed the SEM images of the hydration products and changes of the pore structure of the surface layer of wallboard when the content of *B. mucilaginosus* were 0 and 4%, respectively. In Figure 8A, there were many needle ettringite crystal, Ca(OH)_2_, and some flocculent calcium silicate hydrate gel. The existence of large number of pores contributed to the loose structure of the wallboard. However, from Figure 8B, the pores between hydration products were filled with CaCO_3_ induced by *B. mucilaginosus*, so the structure was more compact. Figure 9 showed the SEM images of the interface transition zone of paste-aggregate of the surface layer when the content of *B. mucilaginosus* were 0 and 4% respectively. In Figure 9A, a gap could be clearly seen between hydration products and aggregate. This phenomenon illustrated that the binding force between them was small and weak. In the later picture Figure 9B, the past and aggregate could combine closely and the structure was more compact. The transformation of the microscopic structure after adding *B. mucilaginosus* could explain the changes of permeability, compressive and flexural strength of wallboard. Three of these properties improved when the content of bacteria was lower than 4%.

## Conclusion

In order to seek an effective way to reduce the efflorescence on the surface layer of wallboard, this study investigated the feasibility of adding *B. mucilaginosus* into mortar and optimal content was determined. The experimental results showed that efflorescence could be reduced greatly by pre-adding *B. mucilaginosus* into mortar and the effect was best when the content of bacteria was 4% of the mass of cementitious material. The results of TGA demonstrated the substantial increase of calcium carbonate and decrease of calcium hydroxide when the content of *B. mucilaginosus* increased from 0 to 4%. The results of permeability, compressive and flexural strength tests also proved that the optimal bacteria content was 4%. The wallboard with *B. mucilaginosus* could be used in more hostile environment, so it could meet the higher requirements. SEM images clearly reflected the decrease of pore numbers and the denser interface transition zone of paste-aggregate of the surface layer when the content of bacteria was lower than 4%. Compared to the traditional method like adding active mineral admixture into cement matrix to reduce the content of Ca(OH)_2_, this method has the advantages of convenient operation, environmental protection (no any harmful material emerging) and low cost. It was suggested that biomineralization might solve the problem of surface efflorescence of wallboard effectively in the near future.

### Conflict of interest statement

The authors declare that the research was conducted in the absence of any commercial or financial relationships that could be construed as a potential conflict of interest.

## Footnotes

^1^JGJ/T70-2009:standard for test method of basic properties of construction mortar.
